# Is SARS-CoV-2 an Etiologic Agent or Predisposing Factor for Oral Lesions in COVID-19 Patients? A Concise Review of Reported Cases in the Literature

**DOI:** 10.1155/2021/6648082

**Published:** 2021-05-18

**Authors:** Shahroo Etemad-Moghadam, Mojgan Alaeddini

**Affiliations:** Dental Research Center, Dentistry Research Institute, Tehran University of Medical Sciences, Tehran, Iran

## Abstract

The pathogenic mechanism of SARS-CoV-2 infection is unclear, and its symptoms vary in different patients. Initial reports of COVID-19 concentrated on pulmonary issues, but with time, additional features such as hyposmia/anosmia, dysgeusia, and skin lesions were added to the list of COVID-19 symptoms. There have been an increasing number of reports on oral cavity lesions in individuals with COVID-19, which might be relevant considering that this location is one of the first sites coming into contact with the virus and that it contains the SARS-CoV-2 receptor. We hereby aim to familiarize practicing head and neck clinicians with the range of oral lesions reported in COVID-19 patients and to critically appraise the most recent data on the role of SARS-CoV-2 in these lesions. We also discuss the ongoing debate on the direct/indirect association of oral symptoms with the disease. COVID-19 cases with simultaneous oral symptoms were extracted from the literature, and articles discussing the role of SARS-CoV-2 in oral lesions were compiled and methodically analyzed. We found approximately 95 COVID-19 patients with a wide range of oral lesions. Based on current evidence, the exact role of SARS-CoV-2 in the development of oral lesions remains unclear. Oral examination of patients is needed to provide adequate cases for analysis to clarify unknown problems related to COVID-19. There is evidence to support both the direct and indirect roles of SARS-CoV-2 in the development of oral lesions. Awareness of the possibility of oral manifestations in COVID-19 is important to clarify the range of disease signs and symptoms.

## 1. Introduction

An unexplained viral pneumonia in a cluster of patients residing in Wuhan, China, soon became known as coronavirus disease 2019 (COVID-19) and rapidly spread to more than 200 countries within 3 months. Severe acute respiratory syndrome coronavirus 2 (SARS-CoV-2) was identified as its responsible pathogen and led to an ongoing pandemic [1].

SARS-CoV-2 is a single-stranded, positive-sense RNA virus, believed to originate from bats. The gold standard for its diagnosis is nucleic acid analysis of respiratory tract samples, through RT-PCR. IgM and IgG are also evaluated for the detection of infected cases and are simpler and more affordable than RT-PCR. Nucleic acid assessment is useful in the early stages of the disease, while IgM and IgG turn positive 10 and 20 days after infection, respectively [1]. SARS-CoV-2 is mainly transmitted through respiratory droplets and direct contact; however, the gastrointestinal tract, saliva, urine, and ocular secretions have also been suggested as potential routes, requiring further investigation [2, 3].

Symptoms of COVID-19 include fever, cough, dyspnea, fatigue, sore throat, headache, myalgia, abnormality in olfactory/gustatory senses, gastrointestinal issues, and less commonly, skin lesions. Common paraclinical findings include bilateral/unilateral infiltrates on chest X-ray [4], ground-glass opacity, and bilateral patchy shadowing on chest CT [2] and increases in neutrophil/lymphocyte ratio, plasma D-dimer, and lactate dehydrogenase, in severe cases [4].

A major concern during a pandemic is the identification of the infectious source. Considering that one of the main sources of infection in COVID-19 is the SARS-CoV-2-positive patient, it is important to know all the symptoms and major and minor manifestations of the disease in order to help obtain an early diagnosis. Based on a considerable number of reports, the oral cavity is one of the extrarespiratory sites that have shown manifestations in COVID-19 patients. Xerostomia, ageusia, and anosmia are among the more recognized symptoms in this location, which might occur before the conventional signs of COVID-19 [3]. However, reports on visible oral lesions in these patients are scattered and mostly in the form of case reports. The objective of the present review is to provide a compilation and analysis of the reported oral manifestations associated with SARS-CoV-2 infection to familiarize practicing head and neck clinicians with the possible oral presentation of COVID-19, whether a direct consequence of the infection or secondary to other causes. We also discuss the ongoing debate regarding the direct or indirect association of oral symptoms with COVID-19. Considering that subjective symptoms such as dry mouth and loss of taste/smell have been extensively reported elsewhere, we concentrate on oral mucosal lesions that could be observed and examined by the physician.

## 2. Search Strategy

A thorough search was performed in PubMed, Scopus, Web of Science, and Google Scholar in a systematic manner using the following search strategy: (covid-19 OR “coronavirus disease 2019” OR sars-cov-2 OR “severe acute respiratory syndrome coronavirus 2”) AND (oral OR mouth OR enanthema OR gingiva OR periodontal OR tongue OR lingual OR labial OR lip) AND (features OR manifestations OR lesions OR symptoms OR co-infection OR co-occurrence OR reactivation). We considered all cases reported up until August 26, 2020, published in any article format (Case Report, Letter to Editor, Original Research, etc.). Even cases presented in tables and figures were extracted and added to the study. Lesions reported in unconfirmed COVID-19 cases were excluded. Full texts of all extracted reports were reviewed, and repetitive cases were omitted.

## 3. Oral Lesions Reported in COVID-19 Patients

Martín Carreras-Presas et al. [5], Chaux-Bodard et al. [6], and Galván Casas et al. [7] were among the first to add the oral cavity to the list of locations with possible COVID-19 involvement. Based on our search terms in the aforementioned databases, twenty-one articles were included in the study after omitting repetitions and applying our selection criteria to the 960 retrieved articles. We identified a total of 34 accounts of oral findings in COVID-19 patients, of which 16 had PCR confirmation and the rest were diagnosed by other means. A description of these individuals is presented in Table 1. There were a total of 14 men and 17 women with an age range of 9–96 years. There was no information on gender in 3 and age in 5 patients. An additional 8 pediatric patients with multisystem inflammatory syndrome associated with COVID-19 were also stated to have oral manifestations early in the disease, but no additional information was provided [22]. Fifty-three COVID-19 hospitalized patients were reported with oropharyngeal candidiasis, again with no detailed information [25]. The addition of these cases results in a total of 95 individuals infected with SARS-CoV-2 with some kind of oral lesion. All intraoral locations, including the tonsillar area, were reported to be involved in different articles. The clinical appearance of the lesions fell into a wide range, the most prevalent being ulcers, petechiae, and erythema. It is noteworthy that there were also reports of 5 cases demonstrating clinical signs/symptoms of COVID-19 and oral lesions, but they did not have laboratory confirmation of the disease and were reported to be “suspicious for COVID-19”. These are merely being descriptively mentioned for historic purposes. Two of the patients were male, 56 and 58 years of age, one of which had asthenia, fever, hyposmia, dysgeusia, and cervical lymph node enlargement for 2 days and complained of sore throat and palatal pain. Examination through teleconference revealed herpetic recurrent stomatitis-like lesions, and the patient reported no previous history of these types of lesions. The other patients also complained of palatal pain and showed multiple small palatal unilateral ulcers with no previous history of herpetic infection. He had hypertension and diabetes and most importantly was isolated at home with his wife who had confirmed COVID-19 [5]. The third patient was a 35-year-old woman who displayed fever, halitosis, bilateral submandibular lymphadenopathy, and a clinical diagnosis of necrotizing gingivitis [26]. Images of enanthemas in two other suspected COVID-19 patients, with no mention of sex or age, were provided as supplemental material and appeared as pustular eruption on the right soft palate and tonsillar area in one and petechiae on the soft and hard palate in the other patient [7].

## 4. Discussion

### 4.1. Pathophysiology of COVID-19

The structural proteins of SARS-CoV-2 consist of spike, membrane, envelope, and nucleocapsid. Attachment of the virus to target cells occurs through the spike, which undergoes cleavage after binding to the angiotensin-converting-enzyme-2 (ACE2) receptor. “Attachment,” is followed by “penetration,” by which the virus enters the host cell; “biosynthesis,” where viral RNA is replicated in the nucleus; “maturation” or production of viral particles, and finally “release” [27].

In advanced disease, SARS-CoV-2 induces exaggerated inflammation and systemic immune activation, which can give rise to a cytokine storm in its most severe form [28]. Lymphopenia occurs following the T-cell attack and innate/acquired immune responses, resulting in impaired lymphopoiesis and exacerbated lymphocyte apoptosis [29].

In severe cases, SARS-CoV-2 causes rampant coagulation activation and depletion of clotting factors, observed as the development of thrombotic complications and microthrombi [29]. Proinflammatory cytokines are responsible for this systemic coagulopathy by stimulating mononuclear cells to produce tissue factor and thrombin. This is supplemented by the generation of plasminogen activator and von Willebrand by directly infected ACE2-expressing endothelial cells [29].

In the cytokine storm, infected ACE2-expressing cells release numerous proinflammatory chemokines/chemoattractants that recruit leukocytes, which in turn produce TNF-*α*, IFN-*γ*, and various interleukins that trigger a proinflammatory cascade with immense cytokine expression. This leads to extensive vascular and organ injury as well as prothrombotic events such as platelet activation, production of reactive oxygen species, and reduction of proteins in charge of vascular protection [29, 30].

ACE2 receptors are the virus's gateway to host cells. Their distribution in multiple tissues contributes to the multisystem involvement of COVID-19 and may support possible routes of virus entry. ACE2 has been reported on endothelial cells, salivary glands, and various epithelial cells, including those of the skin and nasal and oral mucosae. The role of ACE2 in SARS-CoV-2 infection has not been fully determined. ACE2 engagement by the virus and its function may be directly responsible for the diverse tissue involvement of COVID-19 [30].

### 4.2. Controversy on the Actuality of True Oral Manifestations in COVID-19

Following the initial reports of oral lesions in patients with SARS-CoV-2 infection, there has been considerable debate on whether these lesions are true manifestations of the disease or have occurred as a secondary phenomenon due to other factors. Arguments in favor and against the direct association between oral manifestations and COVID-19 have been raised, both of which are predicated on the pathophysiology of SARS-CoV-2 infection.

#### 4.2.1. Rationale in support of Oral Lesions Being a True Result of SARS-CoV-2 Infection

Since the mouth is one of the first sites that come into contact with SARS-CoV-2, it has been hypothesized that oral mucosal lesions may be the first sign of COVID-19 [31], especially considering the multiorgan involvement of the disease. Lack of oropharyngeal examination may explain the paucity of reports on oral manifestations of COVID-19 [20, 32].

The inflammatory reaction known to be associated with COVID-19 has been suggested as a possible explanation for oral lesions. In the case reported by Chaux-Bodard et al. [6], vascular inflammation related to SARS-CoV-2 was proposed to be responsible for the macular erythematous lesion found in their patient. In corroboration, inflammation-induced loss of taste and smell were pointed out to be confirmed symptoms of the disease [31]. Oral ulcerations were reported as an early symptom in 9 patients with pediatric multisystem inflammatory syndrome associated with COVID-19 [22]. Also, in agreement, Ciccarese et al. [19] reported petechiae on the skin, palate, and gingivae of a patient with COVID-19 and severe thrombocytopenia. Hyperinflammation triggered by SARS-CoV-2, followed by a cytokine storm and a prothrombotic state, was suggested to rationalize true COVID-19 lesions in their patient. Sakaida et al. [10] reported drug eruptions in a patient with oral and skin lesions after antibiotic/NSAID treatment which later turned into full-blown COVID-19. There was no fever or flu-like symptoms at the initial diagnosis of drug eruption. A skin biopsy showed deep lymphocytic infiltrations, which were stated as not being typical for drug eruptions. Development of drug hypersensitivity was suggested as not being “incidental” and possibly as a result of cytokine storm and Th17 dysregulation which occurs in COVID-related inflammation [10].

The occurrence of oral lesions in other viral infections comparable to those in COVID-19 has raised the possibility that SARS-CoV-2 may also be capable of inducing oral lesions [33]. Other viral infections such as HSV-1, HSV-2, CMV, and EBV have been tested in COVID-19 patients with oral involvement and were shown to be negative [13, 18]. Following the report of EM-like skin eruptions and oropharyngeal lesions in COVID-19 patients [11], it was emphasized that EM is much more likely to be caused by infectious agents than drug intake [8].

The histopathologic similarity between thrombotic vessels in oral lesions and the pulmonary diffuse thrombotic disease of SARS-CoV-2-infected patients was considered in favor of true oral manifestations. Microscopic observation of epithelial vacuolizations and exocytosis in addition to chronic inflammatory infiltration, necrosis, and hemorrhage in the absence of intubation trauma or other sources of infections was also suggested to be an indication of true lesions [18].

ACE2 is expressed in oral mucosa, particularly in epithelial cells of the tongue [22]. Damage caused by viral entry into vascular and mucosal cells through ACE2 attachment has been used to explain oral erosion [19], lingual pain [31], and inflammatory reactions [17] in COVID-19.

#### 4.2.2. Rationale against Oral Lesions Being a True Result of SARS-CoV-19 Infection

Many agree that emotional stress [31, 32, 34] during lockdown can be the source of herpetic lesions, especially in those with underlying diseases related to a worse outcome [35].

Other explanations for oral lesions in COVID-19 patients include “coinfection” with HSV [31] or bacterial agents like those found in acute necrotizing gingivitis [26], “misdiagnosis” of other viral diseases such as herpes zoster [32, 34] or herpes simplex, “similarity of mild COVID-19 with other infections” [34], “reactivation” of oral HSV-1 [9, 34, 36], “co-occurrence” of herpes zoster and EM-like lesions [36] and COVID-19, “strong association” between herpes zoster and COVID-19 [32], and “overlapping diseases” [34].

Oral manifestations in COVID-19 have also been attributed to adverse effects of treatments [17, 34], use of antiseptic substances applied orally [31], drug-associated immunosuppression predisposing the patient to opportunistic infections [34], systemic health decline leading to secondary lesions [17], poor oral hygiene [31], and traumatic injuries [34]. Lack of synchronous occurrence of some of the reported lesions with the symptomatic phase of COVID-19 has also been used to reject true oral lesions [35].

Absence of oral symptoms in reports from active pandemic epicenters around the world, no self-reported oral lesions by patients on Twitter, and not observing any individuals with oral manifestations by researchers who interviewed COVID-19 patients for taste/smell disorders were also listed as factors against the direct association of SARS-CoV-2 and oral lesions [35].

### 4.3. Coinfection or Co-Occurrence of Other Pathogens in COVID-19

Signs and symptoms indicative of other infectious diseases in COVID-19 may prove to be coinfection or co-occurrence, but their observation deserves attention.

*Viruses.* Varicella-like exanthema is being repeatedly reported in COVID-19 patients [15, 16, 37]. Some have mentioned it as a varicella-like exanthema specific to COVID-19, with absent-mild pruritus, which is in contrast to true varicella [37]. Galván Casas et al. [7] raised the question as to whether SARS-CoV-2 could truly create varicella-like lesions and suggested the concept of coinfection. Others have tested the vesicle fluid of COVID-19 patients using herpesvirus (HV) family microarray PCR and reported combinations of HSVs, EBV, and VZV. Due to the lack of simultaneous testing of SARS-CoV-2, its additional participation was not rejected, but it was suggested that HSV and VZV should be ruled out before considering vesicular lesions as being COVID-19-related [15]. On the other hand, it has been proposed to regard herpes zoster as a sign of latent SARS-CoV-2 infection, even in individuals with none/mild upper respiratory symptoms. The premise was based on the lymphopenia known to accompany COVID-19, which is known to cause impaired antiviral response and promote HV recurrence [15, 16]. Fernandez-Nieto et al. [16] reported negative PCR results of SARS-CoV-2 in the vesicular fluid of 3/15 patients who exhibited recurrent herpes simplex or localized herpes zoster. Both HV and SARS-CoV-2 were also negative in the vesicular fluid of four COVID-19 patients [38].

HSV and VZV have also been considered to accompany oral manifestations of COVID-19 in different capacities [34, 36]. At least 9 cases of oral HSV reactivation have been reported in the literature; however, only some had definitive test results to confirm HV and rule out SARS-CoV-2 [9].

*Bacteria.* Metagenomic analyses of COVID-19 patients demonstrated high reads of bacteria, some of which are involved in oral diseases [26]. The oral microbiome can contribute to bacterial SARS-CoV-2 coinfection of the lungs through mechanical and/or increased ventilation, cough, and poor oral hygiene. The virus-bacteria interaction can lead to modifications in cytokines and lymphocytic responses resulting in exacerbation of lung disease. Meticulous oral health care was suggested, especially in those with severe COVID-19 [39].

*Fungi.* Among 1059 hospitalized patients with confirmed SARS-CoV-2, 53 had confirmed oropharyngeal candidiasis, appearing as pseudomembranous lesions, white plaques, and erythematous areas in the oral mucosa. Exaggerated cytokine release and disturbances in cellular immune response may be responsible for fungal coinfections. The importance of examining fungal infections in COVID-19 patients was highlighted [25].

### 4.4. Overview and General Perspective

Since the COVID-19 outbreak, lesions that were routinely diagnosed before the pandemic are being contemplated differently. For example, a febrile patient presenting with pharyngotonsillitis and numerous posterior oral vesicles and ulcers would have been diagnosed as primary herpes simplex infection, whereas with the current situation and reports of similar lesions in COVID-19, the clinician may reconsider and hesitate before making this diagnosis. General concern about workplace contamination and the possibility of staff infection and viral transmission have further complicated the situation and resulted in a possible overdiagnosis of COVID-19.

Highlighting the lack of oral presentation in COVID-19 was suggested to be important by those opposed to the idea of true SARS-CoV-2-related oral lesions. This was to eliminate unnecessary stress among patients with mere oral symptoms and to help clinicians make accurate treatment decisions when encountering oral ulcers, vesiculobullous lesions, or inflammation. Based on this notion, these types of lesions should not be considered as being related to SARS-CoV-2 infection and be managed routinely [35].

This might be true if we assume that COVID-19 definitely could not be accompanied by oral manifestations. However, there may be merit to the reasons given in favor of true oral lesions in this disease. For example, the relationship between COVID-19-induced inflammation/microthrombi [7] and the abovementioned cases [6, 7, 10, 31] seems logical. Inflammation can both directly cause tissue damage and indirectly pave the way for other issues like increased hypersensitivity. Additionally, the demonstration of ACE2 receptors in the oral mucosa [22] and viral particles in saliva [40] can support or at least raise the possibility of true COVID-19 oral lesions [19, 20]. Some studies have reported oral lesions early in the course of disease [7, 10] or their occurrence without conventional systemic symptoms [6]. Therefore, disregarding them completely may result in missing a positive case leading to infection spread.

Appearances similar to other infections do not take away from the attention that should be given to lesions encountered in COVID-19 and cannot provide definitive evidence to rule out SARS-CoV-2 infection. Moderation should be employed in the diagnostic process, and until definitive information is available on pathognomonic oral lesions of COVID-19, the list of differential diagnoses for an erythematous desquamative ulcerative lesion or purpura on the oral mucosa should also include SARS-CoV-2 infection as a possibility and the patient be advised to practice caution and be tested if necessary.

### 4.5. Hypotheses for Patient Management, Prompting Further Research

SARS-CoV-2 infection remains a public health issue, and to date, there are no definitive or specific treatment modalities for infective individuals, and worldwide vaccination has not been achieved. Therefore, repurposing safe, available, and affordable drugs that target any of the pathways involved in COVID-19 might be helpful as adjuncts in the management of patients with this disease. A few approaches that require further research are proposed.

Proton pump inhibitors reduce stomach acid by forming an irreversible bond to hydrogen/potassium ATPase in gastric parietal cells [41]. They have been suggested as treatment options and conversely as risk factors [42] for COVID-19. However, there are studies that support their inhibitory effects on infectious agents that are frequently found in COVID-19 coinfections like HSV-1/ HSV-2 [43] and *Candida albicans* [44]. Whether the use of these drugs is beneficial or detrimental to COVID-19 patients needs further investigation.

Photodisinfection or antimicrobial photodisinfection therapy is a noninvasive technique that uses light irradiation to excite a photosensitizer molecule into a cytotoxic state [45]. It has been employed for treating oral and other infectious diseases. SARS-CoV-2 exists in saliva and can attach to ACE2 receptors in the oral cavity [22, 40]. The bronchoalveolar lavage fluid of COVID-19 patients has been shown to possess oral pathogens, which can lead to coinfections, highlighting the importance of eliminating oral infections [39]. Photodisinfection might be an option to decrease viral/microbial load in the oral cavity, especially in the initial stages of COVID-19 symptom onset when salivary viral load is highest [40]. This might help reduce transmission of SARS-CoV-2 in addition to decreasing the chance of pulmonary coinfections. Considering that photosensitizers can affect the performance of antimicrobial photodisinfection therapy, evaluating agents like curcumin with anti-inflammatory/antimicrobial activities [45] could be an intriguing idea for further research. Other studies have also highlighted the importance of reducing salivary viral load and suggested the use of mouthwashes, especially in dental settings [46].

Endogenous opioids are compounds with anti-inflammatory and immunomodulatory features [47] suggested as potential safe treatment agents for COVID-19, especially in those accompanied by cytokine storm [48]. However, they can increase susceptibility to some oral infections and have been shown to potentiate HSV-1-induced encephalitis after acute exposure [49]. The applicability of these agents in COVID-19 requires additional investigation.

SARS-CoV-2 spike subunits bind to ACE2 receptors, after which cleavage takes place mainly through transmembrane protease serine 2, but also by other polyprotein convertases, like furin and cathepsin [6]. Adam10 and Adam17 are ACE2 sheddases and are expressed in normal oral epithelium [50] adding to the chance of virus fusion and increased tropism. Their upregulation in oral squamous cell carcinoma [51] might indicate a higher chance of SARS-CoV-2 infection in patients with this cancer. This requires further exploring while taking into account that pharmaceutical blockers have been developed against these sheddases [51].

## 5. Conclusion

To date, a total of 100 COVID-19 patients were reported to have oral lesions, including reactivations, coinfections, and co-occurrences. Based on current data, it is not possible to determine with certainty, whether these lesions are a direct result of SARS-CoV-2 infection or due to other causes like stress, coinfection/co-occurrence, or drug reactions. There are justifications in support of both concepts. At this time, observing oral lesions similar to those reported in COVID-19 patients can neither confirm nor rule out the disease without additional testing. Similarly, considering the possibility of coinfection, even with a definite diagnosis of a non-COVID-19 lesion, we cannot be certain of the absence of COVID-19. Until the pandemic is under control, research on available safe drugs or techniques could be helpful in the introduction of substances that can be used as adjuncts to COVID-19 management.

## Figures and Tables

**Table 1:** Reported oral findings in COVID-19 patients, including cases with reactivation of previous infections*∗*.

Authors/date of submission (2020)	Basis of diagnosis	Sex	Age (y)	Underlying disease s	General/extraoral symptoms	Chief complaint	Oral manifestation	Outcome	Additional information
Chaux-Bodard et al./April 11 [6]	Nasopharyngeal swab on day 8	F	45	NS	(i) Erythematous painful toe lesion, 3 d after oral lesion, painful for 2 d (ii) Mild asthenia		1 d painful inflamed lingual papilla, 1 d red macula, and finally asymptomatic tongue ulcer	(i) Complete healing after 10 d	(i) COVID-19 vasculitis could be responsible for oral macule (ii) Oral/skin lesions were the first signs, and general symptoms were minimal

Martín Carreras-Presas et al./April 20 [5]	Bilateral pneumonia	F	65	Obesity and hypertension, took diuretics and ACE inhibitor	(i) High fever, diarrhea, bilateral pneumonia (ii) Developed rash under breasts, back, and genitalia (3^rd^ week)	(i) Tongue pain from the beginning (ii) Skin rash 23 d later	Ex: blisters on internal labial mucosa and desquamative gingivitis 30 d later	Improvement within 3 d after treatment with mouthwash and prednisolone	Biopsy: some criteria suggestive of viral exanthema or urticarial dermatitis with discrete blood extravasation

Putra et al./April 11 [8]	Oropharyngeal and nasopharyngeal swab, PCR (day 3 of symptoms)	M	29	None	(i) Fever, sore throat, back pain, myalgia, dry cough, 1^st^-3^rd^ days (ii) Lymphopenia and neutrophilia, (2^nd^ day) (iii) Multiple lenticular red papules (3 mm) on extremities with pins and needles sensation (day 3	General symptoms	(i) Aphthous stomatitis, day 7	(i) Skin lesions on day 6 of symptoms, darkening on day 7 (ii) Pins/needles sensation resolve on day 8 (iii) Oral lesions resolve on day 10 (iv) All symptoms gone except dry cough on day 11 (v) Rhinorrhea and anosmia appear on days 12–14	Hand, foot, and mouth disease was rejected based on clinical manifestations and age

Hedou et al./April 21 (accepted) [9]	Nasopharyngeal PCR	NS			Respiratory problems leading to intubation	NS	Oral HSV-1 reactivation during illness	Patient alive	Admitted to intensive care

Galván Casas et al./April 28 (accepted) [7]	Lab confirmation	M	NS		Maculopapular eruptions, other symptoms NS	NS	Desquamative gingivitis-like lesions, petechiae on the lower lip, and erythema on the palate	NS	Appearance of oral lesions is described based on the images provided by the authors and not the authors' statements

Sakaida et al. /April 29 [10]	PCR negative 8 d after oral/skin lesions, turned positive 11 d after oral/skin lesions	F	52	NS	Initially had erythematous skin/oral lesions but after 8 d developed high fever, cough, chills, fatigue, dyspnea, WBC and CRP, lymphopenia, neutrophilia, and opacity in lower lung lobes on CT	Itchy erythema on limbs, erosions on lips, and buccal mucosa	Erosions on lips and buccal mucosa 2 d after antibiotic and NSAID	Transferred to an intensive care unit in another hospital	Skin biopsy showed deep lymphocytic infiltrations, which are not typical in drug eruptions

Jimenez-Cauhe et al./May 5 (accepted) [11]	NS	F	58–77	NS	Erythema multiforme skin lesions	NS	Palatal macules and petechiae	2-3 w after corticosteroid treatment	In at least 2 of them, skin rashes appeared after discharge and their CRP, D-dimer, and lymphocyte count worsened; at least one of them was negative for infectious diseases
		F							
		F							

Aghazadeh et al./May 7 (received) [12]	Nasopharyngeal swab RT-PCR	F	9	None	(i) Weakness, loss of appetite, fever, abdominal pain, diarrhea, and red edematous papules and plaques on dorsal hands and feet (ii) Dry cough, tachypnea, hypoxia, and somnolence	Malaise and oral/skin eruptions	Ex: vesicles, erosions, and herpetiform eruptions on lips, anterior tongue, and buccal mucosa	General symptoms improved in a few weeks, mucocutaneous eruption resolved in about a week	(i) HFMD was rejected due to acral eruption (ii) No targetoid lesion or drug-intake history (iii) PCR for HSV not performed (iv) Oral/skin lesions preceded conventional COVID-19 symptoms

Ansari et al./May 11 [13]	Nasopharyngeal swab PCR	F	56	Diabetes mellitus	Fever and dyspnea	Sores in the mouth on 5^th^ day of general symptoms	Ex: multiple painful ulcers on red nonbleeding background on the entire hard palate	Healing 1 w later, no scarring	(i) Negative HSV1/2 AB (ii) Biopsy: desquamation, edema, granulation, ulceration, mononuclear cell invasion, and neutrophil infiltration secondary to bacteria
	Nasopharyngeal swab PCR	M	75	Hypertension	Hypoxia upon admission	Dysphasia 1 w after admission	Ex: multiple painful ulcers on red nonbleeding background the on anterior tongue		

Askin et al./May 14 [14]	RT-PCR or chest CT	NS	NS				Ex: enanthema and aphthous stomatitis	NS	
		M					Ex: aphthous stomatitis on lateral tongue		
		NS					Ex: rash and enanthema		

Llamas-Velasco et al./May 20 (available online) [15]	Nasopharyngeal swab	F	59	None	Fever, dry cough, dyspnea, bilateral interstitial pneumonia	NS	Vesicles and punched out perioral erosions	NS	Combination of HSV-1, HSV-6, and EBV based on a herpesvirus family microarray PCR of the vesicle fluid

Fernandez-Nieto et al./May 27 (received date) [16]	Nasopharyngeal swab for SARS-CoV-2	M	69	None	Bilateral interstitial pneumonia (ICU admittance)	NS	Orolabial recurrent herpes simplex	NS	Vesicular content: HSV-1 + (multiplex herpes PCR), SARS-CoV-2-(RT-PCR)
		F	96	Hypertension, chronic kidney disease, hyperuricemia	Bilateral interstitial pneumonia				Vesicular content: HSV-1 + (multiplex herpes PCR), SARS-CoV-2-(RT-PCR)
		F	77	Primary biliary cholangitis, Alzheimer	Bilateral interstitial pneumonia				Vesicular content: HSV-1 + (multiplex herpes PCR)
		M	65	Hypertension, dyslipidemia	Bilateral interstitial pneumonia (ICU admittance)				Vesicular content: HSV-1 + (multiplex herpes PCR)
		M	38	Colorectal cancer with chemotherapy	Bilateral interstitial pneumonia				Vesicular content: HSV-1 + (multiplex herpes PCR)
		M	61	None	Bilateral interstitial pneumonia (ICU admittance)				Vesicular content: HSV-1 + (multiplex herpes PCR)
		F	45	None	Bilateral interstitial pneumonia				
		M	76	Hypertension, dyslipidemia	Bilateral interstitial pneumonia				

Amorim Dos Santos et al./May 29 [17]	Nasopharyngeal swab, RT-PCR	M	67	Revascularized respiratory disease, hypertension, ADPKD, and kidney transplant Regular intake of immunosuppressants and use of pharmacological prophylaxis for venous pulmonary thromboembolism	(i) Respiratory symptoms, progressive dyspnea on exertion, fever, and diarrhea (10 d before admission) (ii) Bilateral diffuse hyperdense “ground-glass” infiltrations on chest CT, upon admission (iii) No skin lesions	General symptoms and hypogeusia (admission)	Ex: viscous saliva, persistent white plaque, and pinpoint yellowish ulcers on dorsal tongue similar to late-stage herpetic recurrent lesions (i) Tongue scrape culture showed *Saccharomyces cerevisiae* (24 d postadmission) (ii) Severe asymptomatic geographic and fissured tongue 2 w after drug prescription Photograph: asymptomatic moderate geographic tongue and tonsillar erythema 10 d postdischarge	(i) Lingual white plaque remained after oral nystatin and IV fluconazole in the hospital (ii) Near complete resolution of white tongue lesions after 2 w chlorhexidine, hydrogen peroxide, oral hygiene care, and continuation of antifungals	(i) Received supplemental O_2_ and orotracheal intubation

Soares et al./June 2 [18]	PCR	M	42	Diabetes and hypertension	(i) Fever, cough, shortness of breath (ii) Petechiae -like, vesiculobullous skin lesions (unknown etiology)	Painful ulceration in buccal mucosa	Ex: ulcerations and multiple reddish macules scattered throughout the hard palate, tongue, and lips	Complete resolution of oral lesions after 3 w	Biopsy: epithelial vacuolization, chronic inflammation, focal necrosis, hemorrhage, vessel thrombi, and CD3^+^ and CD8^+^ infiltration of minor salivary glands

Ciccarese et al./June 2 [19]	Nasopharyngeal swab, RT-PCR	F	19	None	(i) Ex: afebrile, red macules, papules, and petechiae on lower extremities (ii) WBC and lymphocytes and thrombocytopenia	Fever/sore throat for 7 d, fatigue, hyposmia, and skin/oral/pharyngeal lesions (day 5)	Ex: erosions, ulcerations, and hemorrhagic crusts on labial mucosae (i) Petechiae on palate and gingiva	(i) Regression of systemic lesions, day 5 after treatment (ii) Complete resolution of oral and cutaneous lesions on day 10	(i) Thrombocytopenia seen from initial stages—petechial lesions probably due to severe thrombocytopenia, triggered by SARS‐CoV‐2, worsened by cefixime

Cebeci Kahraman and Çaşkurlu/June 4 [20]	2 positive rapid COVID-19 IgM tests 1 d apart	M	51	None	(i) Fever, fatigue, dry cough, sore throat, and taste and smell issues	Sore throat, which worsened 10 d after symptom onset	(i) Ex: erythema on oropharynx and hard palate, midline petechiae, soft palate pustular enanthema (border)	Resolved after a few days of antibiotic therapy	(i) COVID-19 IgM^+^ and IgG^+^ after 2 w, (ii) PCR was negative 2 w + 2 d after isolation and all symptoms resolved

Tomo et al./July 2 (received) [21]	Nasopharyngeal swab, PCR	F	37	None	Fever, asthenia, anosmia	Worsening of dysgeusia, burning tongue, and dry mouth on day 9	(i) Dysgeusia, burning tongue, and dry mouth for 3 d (ii) Telemedically (day 9): diffuse erythema and depapillation with red spots on lingual borders, no lesion on the palate	Symptomless. 2 w after COVID-19 onset and treatments	

Cant et al./July 3 (published online) [22]	NS	M	9	Severe dystonia and epilepsy	Fever, malaise, and GI upset	Swollen lip and oral ulcer 2^nd^ time in 2 w, each followed by fever, malaise, and GI upset	Swollen lip and oral ulcerations	Improvement 3 d after hydrocortisone treatment	(i) Eight other children with the same oral lesions before pediatric multisystem inflammatory syndrome associated with COVID-19, in the same unit

Díaz Rodríguez et al./July 22 (accepted manuscript) [23]	PCR	F	43	NS	Fever, malaise, dysgeusia, anosmia, diarrhea, pneumonia, risk of thrombosis, based on lab test	Aphthous-like lesions, burning sensation	Photograph: aphthous-like lesions, progressive tongue depapillation	Disappearance of ulcers and burning 10 d after triamcinolone rinse but not the depapillation	
	Positive for SARS-CoV-2, method not specified	M	53		Hospital admission	Burning mouth, unilateral commissural fissures, anosmia, and dysgeusia	Ex: commissural cheilitis	Angular cheilitis, but not anosmia and dysgeusia, disappeared after antibiotics, nystatin, and hygiene measure	Oral manifestations were found few days after hospital discharge
	Positive for SARS-CoV-2, method not specified	F	78	NS	Hospital admission	Dry mouth sensation	Ex: angular cheilitis and pseudomembranous candidiasis-like lesions on tongue, palate, and lip commissure	Angular cheilitis and pseudomembranous lesions disappeared after nystatin and antibiotics, dry mouth improved after solutions/gels prescription	Symptoms appeared since hospitalization

Glavina et al./August 9 (accepted online) [24]	PCR	F	40	Frequent recurrent herpes labialis eruption	Weakness, fever, and acute loss of taste	Malaise, fever, ageusia, oral pain,andburning7 d after diagnosis	Telemedically: recurrent palatal HSV, white hairy tongue, nonspecific white lateral tongue lesion	Healing after 3 w and double-negative PCR	

NS: not specified; d: day; ACE: angiotensin-converting enzyme; w: week; Ex: examination; WBC: white blood cell; CRP: C-reactive protein; CT: computerized tomography; NSAID: nonsteroid anti-inflammatory drug; HSV-1/HSV-2: herpes simplex virus 1 and 2; AB: antibody; EBV: Epstein–Barr virus; LAP: lymphadenopathy; ADPKD: autosomal dominant polycystic kidney disease; ICU: intensive care unit; GI: gastrointestinal; HFMD: hand, foot, and mouth disease. ^∗^A total of 53 COVID-19 hospitalized patients were reported to have oropharyngeal candidiasis, without detailed information for each individual patient [25].

## References

[1] Harahwa T. A., Lai Yau T. H., Lim-Cooke M. S., Al-Haddi S., Zeinah M., Harky A. (2020). The Optimal diagnostic methods for COVID-19. http://j/dx.ahead-of-print/dx-2020-0058/dx-2020-0058.xml.

[2] Guan W.-J., Ni Z.-Y., Hu Y. (2020). Clinical characteristics of coronavirus disease 2019 in China. *The New England Journal of Medicine*.

[3] Freni F., Meduri A., Gazia F. (2020). Symptomatology in head and neck district in coronavirus disease (COVID-19): a possible neuroinvasive action of SARS-CoV-2. *American Journal of Otolaryngology*.

[4] Ren Y. R., Golding A., Sorbello A. (2020). A comprehensive updated review on SARS-CoV-2 and COVID-19. *The Journal of Clinical Pharmacology*.

[5] Martín Carreras-Presas C., Amaro Sánchez J., López-Sánchez A. F., Jané-Salas E., Somacarrera Pérez M. L. (2020). Oral vesiculobullous lesions associated with SARS-CoV-2 infection. *Oral Diseases*.

[6] Chaux‐Bodard A. G., Deneuve S., Desoutter A. (2020). Oral manifestation of Covid‐19 as an inaugural symptom?. *Journal of Oral Medicine and Oral Surgery*.

[7] Galván Casas C., Català A., Carretero Hernández G. (2020). Classification of the cutaneous manifestations of COVID-19: a rapid prospective nationwide consensus study in Spain with 375 cases. *British Journal of Dermatology*.

[8] Putra B. E., Adiarto S., Dewayanti S. R., Juzar D. A. (2020). Viral exanthem with “Spins and needles sensation” on extremities of a COVID-19 patient: a self-reported case from an Indonesian medical frontliner. *International Journal of Infectious Diseases*.

[9] Hedou M., Carsuzaa F., Chary E., Hainaut E., Cazenave-Roblot F., Masson Regnault M. (2020). Comment on ’Cutaneous manifestations in COVID-19: a first perspective’ by Recalcati S. *Journal of the European Academy of Dermatology and Venereology*.

[10] Sakaida T., Tanimoto I., Matsubara A., Nakamura M., Morita A. (2020). Unique skin manifestations of COVID-19: is drug eruption specific to COVID-19?. *Journal of Dermatological Science*.

[11] Jimenez-Cauhe J., Ortega-Quijano D., Carretero-Barrio I. (2020). Erythema multiforme-like eruption in patients with COVID-19 infection: clinical and histological findings. *Clinical and Experimental Dermatology*.

[12] Aghazadeh N., Homayouni M., Sartori-Valinotti J. C. (2020). Oral vesicles and acral erythema: report of a cutaneous manifestation of COVID-19. *International Journal of Dermatology*.

[13] Ansari R., Gheitani M., Heidari F., Heidari F. (2020). Oral cavity lesions as a manifestation of the novel virus (COVID-19). *Oral Diseases*.

[14] Askin O., Altunkalem R. N., Altinisik D. D., Uzuncakmak T. K., Tursen U., Kutlubay Z. (2020). Cutaneous manifestations in hospitalized patients diagnosed as COVID-19. *Dermatology Therapy*.

[15] Llamas-Velasco M., Rodríguez-Jiménez P., Chicharro P., De Argila D., Muñoz-Hernández P., Daudén E. (2020). Reply to “Varicella-like exanthem as a specific COVID-19-associated skin manifestation: multicenter case series of 22 patients”: to consider varicella-like exanthem associated with COVID-19, virus varicella zoster and virus herpes simplex must be ruled out. *Journal of the American Academy of Dermatology*.

[16] Fernandez-Nieto D., Ortega-Quijano D., Suarez-Valle A., Burgos-Blasco P., Jimenez-Cauhe J., Fernandez-Guarino M. (2020). Comment on: “To consider varicella-like exanthem associated with COVID-19, virus varicella zoster and virus herpes simplex must be ruled out. Characterization of herpetic lesions in hospitalized COVID-19 patients. *Journal of the American Academy of Dermatology*.

[17] Amorim Dos Santos J., Normando A. G. C., Carvalho da Silva R. L. (2020). Oral mucosal lesions in a COVID-19 patient: new signs or secondary manifestations?. *International Journal of Infectious Diseases*.

[18] Soares C. D., Carvalho R. A., Carvalho K. A., Carvalho M. G., Almeida O. P. (2020). Letter to editor: oral lesions in a patient with covid-19. *Medicina oral, patologia oral y cirugia bucal*.

[19] Ciccarese G., Drago F., Boatti M., Porro A., Muzic S. I., Parodi A. (2020). Oral erosions and petechiae during SARS-CoV-2 infection. *Journal of Medical Virology*.

[20] Cebeci Kahraman F., Çaşkurlu H. (2020). Mucosal involvement in a COVID-19-positive patient: a case report. *Dermatology Therapy*.

[21] Tomo S., Miyahara G. I., Simonato L. E. (2020). Oral mucositis in a SARS-CoV-2-infected patient: secondary or truly associated condition. *Oral Diseases*.

[22] Cant A., Bhujel N., Harrison M. (2020). Oral ulceration as presenting feature of paediatric inflammatory multisystem syndrome associated with COVID-19. *British Journal of Oral and Maxillofacial Surgery*.

[23] Díaz Rodríguez M., Jimenez Romera A., Villarroel M. (2020). Oral manifestations associated to Covid-19. *Oral Diseases*.

[24] Glavina A., Biočina-Lukenda D., Mravak-Stipetić M., Markeljević J. (2020). Oral symptoms and lesions in SARS-CoV-2 positive patient. *Oral Diseases*.

[25] Salehi M., Ahmadikia K., Mahmoudi S. (2020). Oropharyngeal candidiasis in hospitalised COVID-19 patients from Iran: species identification and antifungal susceptibility pattern. *Mycoses*.

[26] Patel J., Woolley J. (2020). Necrotizing periodontal disease: oral manifestation of COVID-19. *Oral Diseases*.

[27] Yuki K., Fujiogi M., Koutsogiannaki S. (2020). COVID-19 pathophysiology: a review. *Clinical Immunology*.

[28] Morris G., Bortolasci C. C., Puri B. K. (2020). The pathophysiology of SARS-CoV-2: a suggested model and therapeutic approach. *Life Science*.

[29] Wiersinga W. J., Rhodes A., Cheng A. C., Peacock S. J., Prescott H. C. (2020). Pathophysiology, transmission, diagnosis, and treatment of coronavirus disease 2019 (COVID-19): a review. *JAMA*.

[30] Xu H., Zhong L., Deng J. (2020). High expression of ACE2 receptor of 2019-nCoV on the epithelial cells of oral mucosa. *International Journal of Oral Science*.

[31] Petrescu N., Lucaciu O., Roman A. (2020). Oral mucosa lesions in COVID-19. *Oral Disease*.

[32] de Carvalho L. F. D. C. E. S., Kitakawa D., Cabral L. A. G. (2020). Oral lesions of herpes zoster in COVID-19 patients or truly associated to the disease?. *Oral Disease*.

[33] Vieira A. R. (2020). Oral manifestations in coronavirus disease 2019 (COVID-19). *Oral Diseases*.

[34] Ponce J. B., Tjioe K. C. (2020). Overlapping findings or oral manifestations in new SARS-CoV-2 infection. *Oral Diseases*.

[35] Al-Khatib A. (2020). Oral manifestations in COVID-19 patients. *Oral Diseases*.

[36] Rocha A. L., de Souza A. F., Resende R. G. (2020). Current evidence on possible oral manifestations of SARS-CoV-2 infection. *Oral Diseases*.

[37] Marzano A. V., Genovese G., Fabbrocini G. (2020). Varicella-like exanthem as a specific COVID-19-associated skin manifestation: multicenter case series of 22 patients. *Journal of the American Academy of Dermatology*.

[38] Fernandez-Nieto D., Ortega-Quijano D., Jimenez-Cauhe J. (2020). Clinical and histological characterization of vesicular COVID-19 rashes: a prospective study in a tertiary care hospital. *Clinical and Experimental Dermatology*.

[39] Bao L., Zhang C., Dong J., Zhao L., Li Y., Sun J. (2020). Oral microbiome and SARS-CoV-2: beware of lung Co-infection. *Frontier Microbiology*.

[40] Zhu J., Guo J., Xu Y., Chen X. (2020). Viral dynamics of SARS-CoV-2 in saliva from infected patients. *Journal of Infection*.

[41] Shirazi M., Alimoradi H., Kheirandish Y. (2014). Pantoprazole, a proton pump inhibitor, increases orthodontic tooth movement in rats. *Iranian Journal of Basic Medical Sciences*.

[42] Charpiat B., Bleyzac N., Tod M. (2020). Proton pump inhibitors are risk factors for viral infections: even for COVID-19?. *Clinical Drug Investigation*.

[43] Michaelis M., Kleinschmidt M. C., Bojkova D., Rabenau H. F., Wass M. N., Cinatl J. (2019). Omeprazole increases the efficacy of acyclovir against herpes simplex virus type 1 and 2. *Frontiers in Microbiology*.

[44] Nobile C. J., Ennis C. L., Hartooni N., Johnson A. D., Lohse M. B. (2020). A selective serotonin reuptake inhibitor, a proton pump inhibitor, and two calcium channel blockers inhibit Candida albicans biofilms. *Microorganisms*.

[45] Pourhajibagher M., Partoazar A., Alaeddini M., Etemad-Moghadam S., Bahador A. (2020). Photodisinfection effects of silver sulfadiazine nanoliposomes doped-curcumin on Acinetobacter baumannii: a mouse model. *Nanomedicine (Lond).*.

[46] Imran E., Khurshid Z., M Al Qadhi A. A., A Al-Quraini A. A., Tariq K. (2020). Preprocedural use of povidone-iodine mouthwash during dental procedures in the COVID-19 pandemic. *European Journal of Dentistry*.

[47] Doustimotlagh A. H., Dehpour A. R., Etemad-Moghadam S., Alaeddini M., Kheirandish Y., Golestani A. (2017). Nitrergic and opioidergic systems affect radiographic density and histomorphometric indices in bile-duct-ligated cirrhotic rats. *Histology Histopathology*.

[48] Tahamtan A., Tavakoli-Yaraki M., Salimi V. (2020). Opioids/cannabinoids as a potential therapeutic approach in COVID-19 patients. *Expert Review of Respiratory Medicine*.

[49] Eisenstein T. K. (2019). The role of opioid receptors in immune system function. *Frontier Immunology*.

[50] Gupta I., Rizeq B., Elkord E., Vranic S., Al Moustafa A. E. (2020). SARS-CoV-2 infection and lung cancer: potential therapeutic modalities. *Cancers (Basel)*.

[51] Etemad-Moghadam S., Alaeddini M. (2019). Upregulation of ADAM10 in oral squamous cell carcinoma and its correlation with EGFR, neoangiogenesis and clinicopathologic factors. *Journal of Cranio-Maxillofacial Surger*.

